# Supraclavicular Artery Flap for Head and Neck Oncologic Reconstruction: An Emerging Alternative

**DOI:** 10.1155/2013/658989

**Published:** 2013-12-29

**Authors:** Ashok Shenoy, Vijayraj S. Patil, B. S. Prithvi, P. Chavan, Rajshekar Halkud

**Affiliations:** ^1^Department of Head and Neck Surgery, Kidwai Memorial Institute of Oncology, Bangalore, Karnataka 560029, India; ^2^Department of Surgical Oncology, HCG Hospital, Bangalore 560027, India

## Abstract

*Aim*. Head and Neck oncologic resections often leave complex defects which are challenging to reconstruct. The need of the hour is a versatile flap which has the advantages of both a regional flap (viz. reliable and easy to harvest) and a free flap (thin, pliable with good colour match). In this a study we assessed the usefulness of the supraclavicular artery flap in head and neck oncologic defects. *Materials and Method*. The flap was used as a pedicled fasciocutanous and was based on the transverse supraclavicular artery. We assessed this reconstructive option for complications as well as its and functional out comes. *Results*. Eleven cases underwent supraclavicular artery flap between 20011-2012 of which 5 were males and 6 females. Mean defect size was 5 cm × 6 cm. Nine donor sites were closed primarily and 1 required split skin grafting. We encountered one complete flap loss which was attributed to a band of constricting skin bridge over the vascular pedicle in a defect involving lateral third of midface. Two patient developed pharyngeocutaneous fistula (without flap loss) out of 3 patients who underwent augmentation pharyngoplasty post Near total laryngectomy. *Conclusion*. Supra clavicular artery flap is a thin versatile, reliable, easy to harvest, with good cosmetic and functional outcome at both ends (recipient and donor) for reconstructing head and neck oncologic defects.

## 1. Introduction

Head and neck oncologic resections leave complex defects which are challenging to reconstruct. The various reconstructive options available for resurfacing of this region range from split thickness skin grafts and ultrathin flaps to local or free flaps. In the facial region, one has to take account of the aesthetic units and provide an appropriately thin flap to restore both form and function. The color and texture match is also equally important. A locoregional flap should also leave a minimum of donor site morbidity and preferably be hidden beneath the clothing. According to Gillies' concept, the more adjacent the donor site is, the better the skin will match the recipient site (1). Regional muscle flaps namely pectoralis major myocutaneous flap owing to its bulk is difficult to handle and inset into a 3 diemensional defect; additionally in females the bulk due to breast tissue complicates intraoperative handling and insetting. Further the resultant breast deformity is a source of concern to young women. In males this is a relatively less hairy zone than the anterior chest in males and can therefore be exploited for interior mucosal lining. Free flaps need special microvascular expertise, added operating time in patients and increase costs due to prolonged hospital stay and operating theater time. The need of the hour is a flap which has the advantages of both regional flap (reliable and easy to harvest) and free flap (thin, pliable good colour match).

Supraclavicular artery flap is a fasciocutaneous flap based on supraclavicular artery, a branch of the transverse cervical artery, less frequently it arises from, the suprascapular artery, though reliable, is not a very large vessel. Venous drainage is usually accompanied by transverse cervical vein which should be identified at its upper extent by carefully preserving external jugular vein which drains the distal portion of the flap.

In this study (2011-2012) we recount our experience with respect the utility of supraclavicular artery flap in head and neck oncologic defects in 11 consecutive cases done by senior author (AMS).

## 2. Materials and Method

After taking informed consent from patients, this report is a prospective study of cases who underwent supraclavicular artery flap between 2011 and 2012 of which 5 were males and 6 females. Cases included 8 mucosal lining reconstruction, namely, resection palate (1case), inferior alveolus after marginal mandibulectomy (1), buccal mucsa resurfacing following composite resection for buccoalveolar cancer (1), full tubed hypopharyngeal defect after circular pharyngectomy (2), partial hypopharyngeal defect with near total laryngectomy (2), and 4 cervicofacial skin defects (2 from parotid composite defects, 1 after post auricular skin reconstruction following temporal bone and 1 cheek defect post oral cancer). Almost all reconstructions were executed on untreated malignant tumors except 1 which was a salvage case for through and through cheek defect following radical radiotherapy. The skin island was designed to fit the resultant defect and was based on the transclavicular vascular pedicle in all cases and almost all donor sites could be closed primarily after undermining.

The flap design was as follows: the outline of the flap was centered over the deltoid-acromial prominence with the size of the defect being designed lateral to the anterior border of the Trapezius muscle; the pedicle length which decided arc of transposition is calculated from this point. All the patients were counseled preoperatively about a visible scar over the donor site and the possibility of stretching of the scar postoperatively was explained. The procedure was performed under general anesthesia with endotracheal intubation.

### 2.1. Technique of Flap Harvest

**Figure 1:**
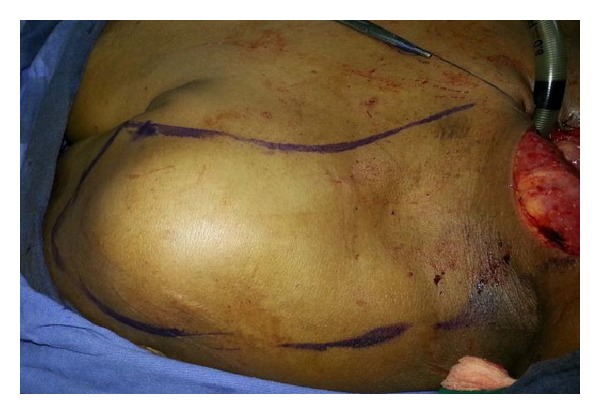
Flap design posteriorly 2 cm above and parallel to scapular spine, anteriorly parallel to clavicular margin laterally parallel to deltopectoral groove.

**Figure 2:**
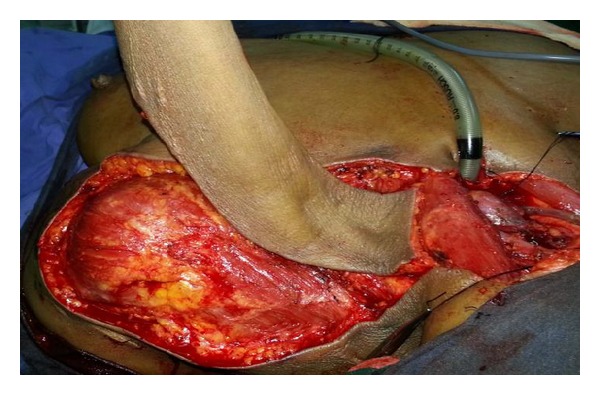
Intraoperative photograph showing flap harvest with pedicle. Note preservation of continuity of medial aspect of pedicle over the triangle, which is not dissected off supraclavicular fat which stays stable attached to the scalene group of muscles at deeper level. Also note completed transection of skin paddle over the sternocleidomastoid which is well visualized.

Patient was placed in supine position with bags under shoulder and ipsilateral hand is extended. Entire neck, axilla, and shoulder region are prepared with betadine and draped. It is important to ascertain absence of metastasis at Level 4 and lower level 5 at the end of the resection or by preoperative evaluation. Presence of significant nodes here will not permit execution of this flap. A contingency PMMF is also highly possible if this instance is detected intraoperatively. Provided that there are no nodes in supraclavicular and posterior triangle, (which is bound anteriorly by posterior border of Sternocledomastoid, posteriorly by anterior border of trapezius and inferiorly by the clavicle is marked) (Figure 1). Pedicle of supraclavicular artery flap lies in this triangle deep to the belly of the omohyoid parallel to the clavicle. In the present study, no hand-held Doppler has been used in any of the cases. Flap outline is marked posteriorly 2 cm anterior to spine of scapula, anteriorly a line parallel to posterior line in front of clavicle, while lateral margin can be extended 2 cms lateral to deltopectoral groove. Flap is elevated from distal to proximal in subfascial plane avoiding damage to pedicle. The communicating perforators from the deltoid branch of the thoraco-acromial axis and posterior circumflex humeral artery are sacrificed. The flap is raised at a subfascial level just superficial to deltoid muscle by sharp knife dissection (avoid monopolar cautery/hot knife to reduce thermal damage) taking care to continuously irrigate with saline as it is elevated proximal towards its pedicle which is visualised all along the anterior border at trapezius. In the upper neck above the apex of posterior triangle plane of elevation is subplatysmal, but this is better avoided in lower part of the neck overlying the triangle. Extreme caution must be exercised not to raise the fibrofatty pedicle in which lie the vessels an ascending branch that traverses upwards parallel to anterior border trapezius and deep branch which pierces deep to the trapezius parallel to the lateral third of the clavicle. The flap is free to be transposed into the defect which can range from the lateral skull base for subtotal temporal bone defects to intraoral defects. Special care is taken not to elevate the pedicle with its fatty adipose tissues from the floor of posterior triangle (Figure 2). Near the point of exit of the supraclavicular vessels, the dissection is done preserving a fascial pedicle of about 3–5 cm in width. The raised flaps are observed for bleeding from the distal end to ensure intraoperative flap viability. The length of the subcutaneous pedicle depends on distance from the medial edge of the defect medial to the lateral third of clavicle where muscle of trapezius insert. The flap is tunneled below the cervical incision along an arc of 120–180 degrees. The area of the pedicle that has to be buried under skin flaps are de-epithelized preserving the subcutaneous over the superficial aspect of the fat flap in entirety. Arc of rotation can be increased by excising carefully the epithelial component sans subcutaneous fat paddle between anterior edge of the trapezius, posterior edge of the sternocleidomastoid and reflecting the clavicular periosteal lining by a Freer elevator on its anterior, superior, and inferior aspects which are then cross-hatched to gain 3 cms additional length; if still there is need for additional mobility then one can transect the sternal and clavicular heads of the sternocleidomastoid muscle flush with the clavicle and manubrium elevating it as a fasioareolar pedicle flap whose base in the triangle is left undisturbed to confer protection to the vessels from tension/torsion. Such a fully mobilized flap is transposed into defect; intervening skin not needed for closure of defect is de-epithelized. The inset is done in two layers: subcutaneous with absorbable 3-0 polygalactin 910 (vicryl) and skin with nonabsorbable 3-0 nylon (ethilon) sutures. A suction drain was used to drain the flap and the donor site, which was removed by the third postoperative day. Primary closure of donor site is done after undermining the chest and skin over scapula both anteriorly and posteriorly, respectively. If there is difficulty in approximating the flaps over acromion closure by split skin grafting is done.

Postoperatively flap was monitored for complications such as flap viability, hematoma, avoiding constricting skin bridges, constricting bandages to prevent vessel compression, and finally avoidance of excessive low temperature in the intensive care unit as well as hypotension.

## 3. Representative Case Description of Different Defects

### 3.1. Case 1: Parotidectomy Defect

A 28-year-old female with adenoid cystic carcinoma of parotid with skin infiltration under parotdiectomy had defect measuring 4 cm × 6 cm. underwent supraclavicular flap reconstruction for skin cover and to prevent retromandibular hollow (Figure 4).

### 3.2. Case 2: Palatal Defect

A 50-year-old female with carcinoma of junction of hard and soft palate underwent wide local excision via lower cheek flap and midface degloving resulting in a palatal defect of 5 cm × 6 cm defect was closed with supraclavicular flap tunneled under mandible (Figure 5).

### 3.3. Case 3: Pharyngeal Defect

A 50-year-old male with carcinoma pyriform fossa after resection had defect of 4 cm × 6 cm underwent pharyngoplasty with supraclavicular artery flap. Postoperatively a pharyngocutaneous fistula developed which responded to conservative therapy (Figure 6).

## 4. Results

**Figure 3:**
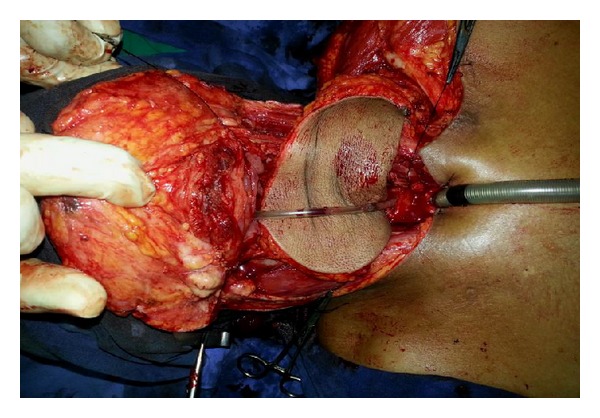
Intraoperative image showing the flap after inset in the defect resulting from circular pharyngectomy of hypopharyngeal cancer. Tumor-free oesophageal and oropharyngeal stumps are confirmed by frozen section intraoperatively.

**Figure 4:**
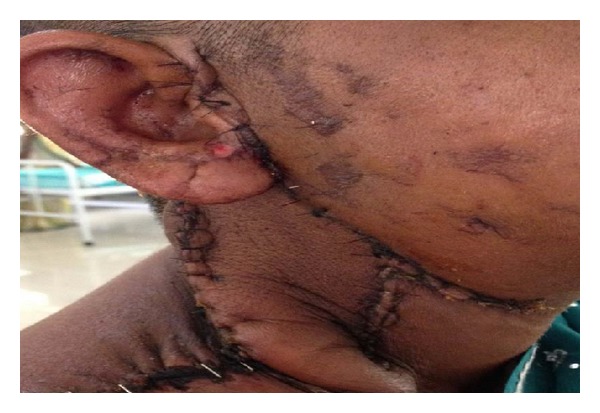
Supraclavicular flap for radical parotidectomy defect.

Eleven cases underwent supraclavicular artery flap between 2011 and 2012 of which 5 were males and 6 females (Table 1). Mean age group is 40–60 years. The surgical defect size ranged from 15 cms to 8–5 cm (pharynx was lined in 8 and cervicofacial skin in 3 with 1 failure in each group −1 total and 1 partial loss). Mean flap dimension was 7 cm × 12 cm (flaps needed for circular pharyngectomy defects Figure 3—approximately 15 cms (length) × 7-8 cms below at esophageal stump and 12–15 ms at oropharynx stump were distinctly larger than those lined pharynx by 20 sq cms). De-epithelization of intervening pedicle was undertaken in all those resurfaced oral-pharyngeal cavity and 1 of the 3 that were used for skin defects. Mean harvesting time was 50 mintues (range 40–62 minutes) and in-setting time took from 20 minutes in small defects to 70 minutes in full pharyngeal tubing. Nine donor sites were closed primarily and 2 required split skin grafting. In the absence of complications the hospital stay ranged from 5 days (skin) to 9 days (inner lining).

**Figure 5:**
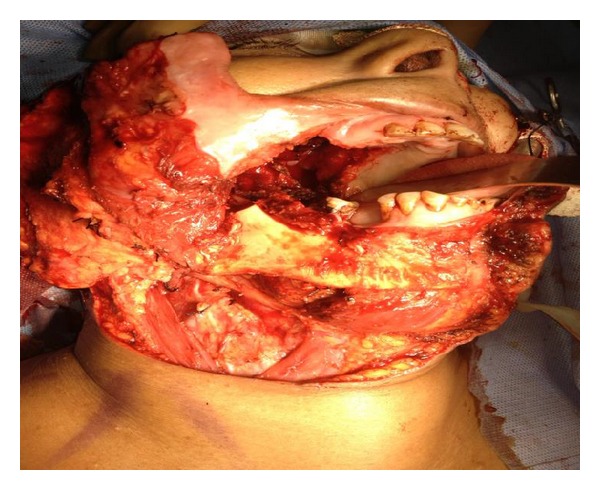
Showing defect after resection of tumor at junction of hard and soft palate.

**Figure 6:**
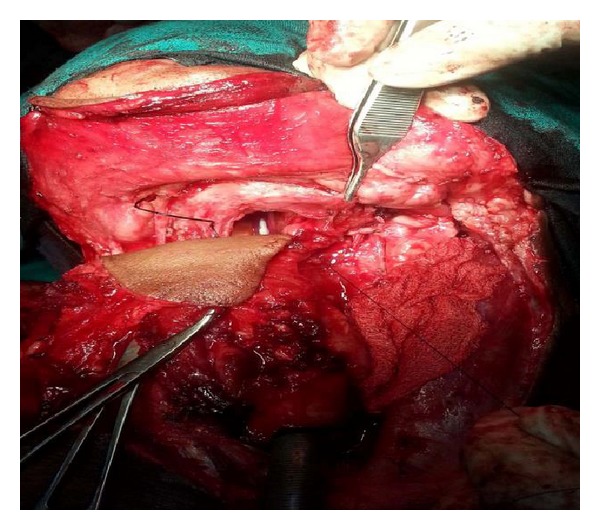
Supraclavicular flap reconstruction of defect after near total laryngectomy and partial pharyngectomy.

**Figure 7:**
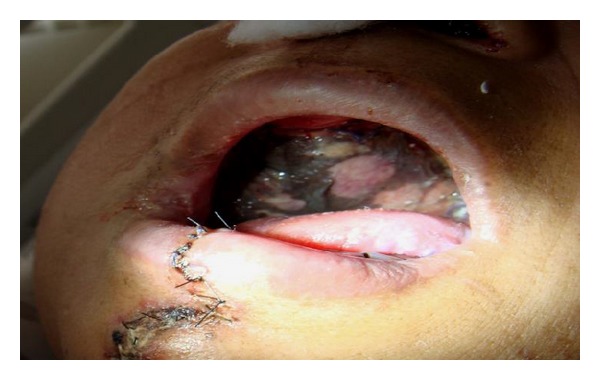
Patient with epidermolysis of flap following hemipalate replacement (Figure 5 case) by TS flap.

We encountered one complete flap loss (parotid region) which was debrided and resurfaced with split thickness graft. Two patients developed pharyngocutaneous fistula out of 3 patients who underwent pharyngoplasty with the island skin remaining viable and dehiscence prevailing at mucosa-skin junction. One required formal repair with a PMMF flap for outer cover as well nearly 3 weeks after the failed flap-mucosa dehiscence while the second was managed by secondary suturing with successful outcome. Other minor complications encountered were prolonged serous (lymphatic) discharge from neck wound (1) and epidermolysis and partial failure of flap that resurfaced palate (Figures 5 and 7) (1). No functional shoulder morbidity was seen.

## 5. Discussion

Toldt [1], an anatomist, in an article cited by Gillies 1923 was the first to illustrate and name the vessel arteria cervicalis superficialis which originated as a branch of thyrocervical trunk. In 1949, the first clinical application of a flap from the shoulder (“charretera” or acromial flap) was performed by Kazanjian and Converse [2]. Charretera, in Spanish, means the shoulder area where honors are bestowed on military personnel. In 1979, the first anatomical studies were performed by Mathes and Vasconez, who described the vascular territory and clinical applications in head and neck reconstruction [3]. The flap was renamed the cervicohumeral flap. The supraclavicular fasciocutaneous island flap was actually introduced by Lamberty and Cormock in 1979. He correctly described the supraclavicular artery as a perforator that arises from the transverse cervical artery in 93% of cases or from the suprascapular artery in 7% of cases [4]. In the beginning of 1990s, Pallua et al. “rediscovered” this flap and popularized its use by performing detailed anatomical studies examining the vascularity of what is known today as the supraclavicular island flap [5–7]. Chiu et al. used for variety of head and neck oncologic defects [8].

Mean harvest time of supraclavicular artery flap was 50 minutes in our study, similar to that seen in Chiu et al. which was less than 1 hour [8]. Mean harvest time in radial free forearm flap is 76 min [9] and additional time for microvascular anastomosis prolongs surgery compared to supraclavicular artery flap. No handheld Doppler was used to trace vascular pedicle. When there is nodal neck metastasis at Level 4 and 5, this procedure may not be warranted in interest of oncologic safety—especially when the primary lesion is located in the hypopharynx. The pedicle should not be tunneled under constricting bands of overlying neck skin when it is used to resurface defects above the mandible. Torsion of the pedicle should similarly be avoided. These 2 factors were responsible for the one and only total flap loss for a post-parotidectomy defect.

Pharyngeal fistula after closure of pharyngeal defects was observed in 2 out of 3 patients, one was minor and was managed conservatively, while the other required a formal PMMF, Shah et al. [10] described the increased loss of cutaneous paddle and leak rates whenever the PMMF flap is inset for inner mucosal defects. No long-term complication was seen on followup. Similar leaks rates were seen in Chiu et al. 3 out of 9 cases. Fistula rates after radial free forearm flap have been reported to be 32% [9] and pectoral major myocutaneous flap has been 13–63% [10, 11]. So with similar fistula rates supraclavicular artery flap avoids need of specialized microvascular expertise and associated ease of insetting to resurface both mucosal defects and outer cutaneous defects due to its pliable nature (with respect to bulkiness of pectoral major flap especially in women and growth of hair when taken in the males). Epidermolysis and partial flap failure was seen in one patient who was asthmatic and was on steroid treatment postoperatively for her asthmatic condition. Patient was treated conservatively and needed an unplanned obturator prosthesis to seal oronasal communication.

Donor site morbidity of supraclavicular artery flap in our study was minimal except for 2 patients requiring split skin grafting. While Chiu et al. reported 2 cases of shoulder cellulitis and 1 shoulder wound dehiscence [8], its to be noted that Chiu et al. did not use routine drain placement below the flap. In our study, such complications were not encountered as we routinely used drains. As there is extensive undermining of flaps anteriorly and posteriorly there is increased chances of seroma formation postoperatively; suction drains prevent and help in reducing wound complications.

Donor site morbidity of pectoral major flap includes loss of anterior axillary fold, distortion of breast form in females, and minor functional deficit due to loss of muscle.

Donor site morbidity of radial free forearm flap includes need of skin graft to close donor area, tendon injuries, reduced strength of grip power, and sensory disturbances [9].

Supraclavicular artery flap has no major cosmetic or functional morbidity compared to traditional pectoral major or radial free forarm flap while concealing donor site by Indian type of dressing especially in females. Now with reports of including middle supraclavicular nerve with flap a sensate flap can be constructed which may add to growing popularity in use of flap [12]. This should be balanced against recent experience of senior author of 2 instances of dysesthesia in a series of 6 total tubing after circular hypopharyngectomy which will feature in an update of a larger series (AMS).

## 6. Conclusion

Supraclavicular artery flap is a thin and pliable, versatile, reliable, and easy to harvest, with good cosmetic and functional outcome at both recipient and donor sites for one stage reconstruction of complex head and neck oncologic defects. It is an excellent alternative to traditional regional and free flaps and has great potential for becoming the gold standard for reconstruction of soft tissue defects in head and neck.

## Figures and Tables

**Table 1:** Defect size/site and outcomes of patients in study.

Defect (*N* = 11)	Number of patients	Mean age	Mean size of defect (in cm)	Complications	Management of complication
Parotidectomy defects	2	45	4 × 5	Flap loss complete (1)	Split skin graft
Floor of mouth defect	2	52	4 × 5	Serous discharge neck wound	Conservative
Defect in palate and bucal mucosa	1	53	5 × 6	Epidermolysis and partial flap failure (1)	Conservative
Temporal bone resection	1	34	4 × 6	—	
Pharyngoplasty circular (Figures 1–4)	2	54	12–15 cms (length) × 6–10 cms (circumference)	Pharyngocutaneous fistula (1),	1 required PMMC flap closure 1 conservative
Cervicofacial skin defect	1	48	5 × 6	Nil	Nil
Patch pharyngoplasty POST NTL (Figure 5)	2	49	3 × 7	FISTULA (1)	Sec. suturing
